# Formulation of a Traditionally Used Polyherbal Product for Burn Healing and HPTLC Fingerprinting of Its Phenolic Contents

**Published:** 2016

**Authors:** Shirin Fahimi, Seyed Alireza Mortazavi, Mohammad Abdollahi, Homa Hajimehdipoor

**Affiliations:** a *Traditional Medicine and Materia Medica Research Center and Department of Traditional Pharmacy, School of Traditional Medicine, Shahid Beheshti University of Medical Sciences, Tehran, Iran.*; b *Department of Pharmaceutics, School of Pharmacy, Shahid Beheshti University of Medical Sciences, Tehran, Iran. *; c *Department of Pharmacology and Toxicology, Faculty of Pharmacy and Pharmaceutical Sciences Research Center, Tehran University of Medical Sciences, Tehran, Iran.*

**Keywords:** Burns, HPTLC fingerprinting; Iranian Traditional Medicine (ITM), *Malva sylvestris*, * Rosa damascene*, *Solanum nigrum*

## Abstract

Nowadays, plants have been considered as powerful agents for treatment of disorders regarding to their traditional use. In Iranian Traditional Medicine (ITM), plants have a special role in the treatment of various diseases. Burns with their devastating outcomes have been discussed in ITM as well. In the present study, a polyherbal ointment (PHO), retrieved from ITM, was formulated for burn healing and it’s HPTLC fingerprint was prepared. Aqueous extracts of *Malva sylvestris* and *Solanum nigrum* leaves and oily extract of *Rosa damascena* petals (4.85%, 4.85% and 33%, respectively) were added to white beeswax, eucerin and white petrolatum as ointment base. In addition to the microbiological tests, physical stability and rheological behavior of the product were assessed. Fingerprinting of phytochemical constituents of PHO was performed by using silica gel plates and toluene: ethyl acetate: acetic acid (60:40:1) and ethyl acetate: formic acid: acetic acid: water (100:11:11:10) as mobile phases. The results showed that PHO was stable towards physical changes and successfully passed microbiological tests. Moreover, PHO exhibited plastic behavior which is in favor of a topical burn product. In addition, HPTLC fingerprinting of PHO demonstrated the presence of several phenolic constituents corresponding to the plant extracts. Regarding to the role of phenolic compounds in wound healing process, PHO could be an appropriate candidate for burn healing with respect to its traditional use in ITM. Moreover, HPTLC fingerprinting could be utilized as an applicable method for quality control of the prepared formulation.

## Introduction

Plants have been considered as potential agents for prevention and treatment of disorders in recent years. Herbal products are largely preferred to synthetic drugs due to their widespread availability as well as the vast empirical and accessible data regarding to their traditional use. However, modern scientific methods should be applied to validate the claims about the therapeutic effects of the plants, resulting in confirmation the traditional system of medicine (1). In Iranian Traditional Medicine (ITM), plants have been used to combat various diseases and pathological conditions. Burns which are one of the most common and devastating forms of trauma (2) have been considered in ITM as well (3). Several ITM prescriptions have been recommended for burn healing in the form of ointments (3-5). Ointments, traditionally named “*Marahem*”, are one of the most ancient pharmaceutical dosage forms used in ITM. Many Iranian traditional scholars believed that "Hippocrates" was the first who invented this topical dosage form in order to combine two medicinal components with different therapeutic effects for a single target. Based on ITM manuscripts, ointments are a combination of fine and pulverized drugs with beeswax, oils, animal fats or bone marrows which were topically used for injuries, wounds and some swellings. It has been believed that beeswax helps to keep the consistency of the ointments which can prevent flowing of the formulation and lead to a long-term durability of the ointment on the skin (6). Oils or oily extracts were considered as one of the most popular components of the ointments in ITM. These dosage forms, traditionally known as “*Adhaan*”, are natural herbal medicines which have been used in ITM to treat various diseases via topical, oral and even nasal routs. Basically, medicinal oils were prepared by direct or indirect methods; In direct approach, the oils were obtained via compression of oil-bearing parts of the plants or distillation of aromatic plant components; while in the latter method, soft and fragrant aerial parts such as flowers, leaves or fleshy fruits were macerated in almond, sesame or olive oil and exposed to the sun or an artificial heat source for several days (7). In addition to the therapeutic effects of the oils, keeping the durability of the ointments on the target organ and reducing adverse effects of the drugs used in the formulation were the most important reasons for their traditional application in ointment formulations (6). Sesame oil is one of the most common oil bases used in ITM for preparation of medicinal oils due to its long-term stability. It has been claimed that topical application of this oil can be useful in burns and warm swellings (8-10).

Among various burn healing prescriptions in ITM, a combination of aqueous extracts of *Malva sylvestris* and *Solanum nigrum* leaves and oily extract of *Rosa damascena* petals has been frequently used (3-5). *Malva sylvestris* L. (Malvaceae), has been traditionally used to treat skin disorders and injuries (11). It has been claimed that the leaves of the plant have powerful anti-inflammatory, antioxidant and anticancer activity and could be a skin tissue integrator (12). According to ITM manuscripts, *M. sylvestris* L. has cool and wet temperament. ITM scholars believed that the leaves of this plant are astringent, desiccant and swelling reliever which could be useful for burn healing (8,13). According to the claims retrieved from oriental medicine, *Solanum nigrum* L. (Solanaceae) has been specially utilized for treatment of inflammation and edema (14). It has been traditionally believed that the plant demonstrated healing effect in burns and infections (15). ITM physicians declared that aqueous extract of* S. nigrum* leaves with its cool and dry temperament, has astringent and restraint effect, so it has been used as a swelling reliever in ITM burn prescriptions with *M. sylvestris* or other ingredients (3, 4, 8, 9). The therapeutic effects of *Rosa damascena* Mill. (Rosaceae), one of the most important *Rosa *species in Iran, have been mentioned in ancient medical books. In addition to perfumery applications of the essential oil of *R. damascena*, it also has exhibited medicinal properties such as analgesic and anti-inflammatory effects (16, 17). Moreover, the essential oil of *R. damascena* has shown antibacterial, antioxidant and skin wound healing properties (16). The most common application form of* R. damascena* in ITM was its oily extract, traditionally named “*Dohn*^,^*ul*^,^*vard*”, which was prepared by macerating the petals of the plant in sesame or olive oil (18). It has been claimed that the oily extract of *R. damascena* has cooling effect as well as desiccant, flesh-growing, antiseptic and analgesic activity and has been used with *M. sylvestri*s, *S. nigrum* or other ingredients for burn wound healing (3, 8, 9). 

In general, the above mentioned herbs are good candidates for burn healing. Therefore, in the present investigation, a topical polyherbal product consisting of *M. sylvestris* and *S. nigrum *leaves aqueous extracts and *R. damascena* petals oily extract was prepared in the form of ointment. Regarding to the importance of preserving the quality of herbal products (19, 20), HPTLC fingerprinting of PHO has been performed to develop a method for quality control of the prepared formulation.

## Experimental

*Plant materials*

The flowers of *Rosa damascena* Mill. were collected from Kashan, Isfahan Province, Iran in April 2011 and the leaves of. *Malva sylvestris* L. and* Solanum nigrum* L. were collected from Shahriyar, Tehran Province, Iran in October 2011. The plants were authenticated in the Herbarium of Traditional Medicine and Materia Medica Research Center (TMRC), Shahid Beheshti University of Medical Sciences, Tehran, Iran. Voucher specimens of *Rosa damascena* Mill. (No. 3378), *Malva sylvestris* L. (No. 3377) and *Solanum nigrum* L. (No. 3375) have been deposited in the Herbarium of TMRC. The plants were air-dried, powdered and stored separately in well-closed containers.

*Chemicals*

Hide powder was purchased from Sigma-Aldrich, UK. Sesame oil was obtained from Henry Lamotte, Germany. All the solvents, reagents and HPTLC silica gel 60 F_254 _plates (20 × 20 cm) were prepared from Merck, Germany.

*Analysis of plant materials*

The plants were analyzed according to their monographs (21, 22) and their total phenolic contents and tannins were determined by using Folin-Ciocalteu reagent and hide powder according to British Pharmacopeia with some modifications (21). Briefly, the appropriate dilution of the aqueous plant extract was oxidized with Folin-Ciocalteu reagent and then the reaction was neutralized with aqueous solution of sodium carbonate (29%, w/v). After 30 min the absorbance of the resulting blue color was measured at 760 nm using water as compensation liquid to obtain total polyphenolic contents. Determination of tannins was conducted in continuation of the above mentioned method by mixing the same dilutions of the aqueous plant extracts with hide powder to separate tannins from other polyphenols. After shaking vigorously for 60 min, the mixtures were filtrated and the above colorimetric procedure was conducted on the filtrates to determine the contents of polyphenols which were not adsorbed by hide powder. Tannin contents of the solutions were calculated with the following equation:

Content of the tannins (mg/g) = [Total phenolics content (mg/g)–Non-adsorbed polyphenols content (mg/g)]

Quantification was done on the basis of the standard curve of pyrogallol. Results were expressed as milligram of pyrogallol equivalent per one gram of dried plant powder. All measurements were made at room temperature in triplicate.

*Preparation of the plant extracts and determination of their total phenolic and tannin contents*

The powdered leaves of *M. sylvestris* and *S. nigrum* were extracted by using decoction method for 30 min (plant: water 1:20 w/v). The extracts were filtered and concentrated under reduced pressure (concentration ratio 100:5). The powdered petals of *R. damascena* was extracted by using sesame oil as solvent (plant: oil 1:5 w/v). The extraction procedure was done in an incubator at 40 ± 1 ºC for five weeks. Every week, the old herbal powder was replaced with the new one.

Total penolics and tannin contents of the extracts were determined by Folin-Ciocalteu reagent and hide powder as described before (21). For oily extract of *R. damascena*, the colorimetric procedure was conducted on the methanol fraction of the oil. The results were expressed as milligram of pyrogallol equivalent per one milliliter of plant extracts. All measurements were made at room temperature in triplicate.

*Formulation of an optimum base for polyherbal product*

In order to obtain a suitable base for polyherbal topical ointment, white petrolatum, white beeswax and eucerin were selected and several experimental formulations with different composition of the mentioned base ingredients were prepared (Table 1). Sesame oil and water were applied in the experimental formulations instead of the oily and aqueous plant extracts (33% and 9.7%, respectively). Butylated hydroxy toluene (BHT) was added to the formulations as antioxidant (0.04%) and methyl and propyl parabens were used as preservatives (0.2% and 0.06%, respectively). The formulations were prepared by fusion method (23).

**Table 1:** Composition of base ingredients in the experimental formulations (%w/w).

**Formulation code**	**Eucerin**	**White petrolatum**	**White beeswax**
**F**_1_	24	26	7
**F**_2_	24	29	4
**F**_3_	25	28	4
**F**_4_	25	29	3
**F**_5_	25	30	2
**F**_6_	29	24	4
**F**_7_	29	25	3
**F**_8_	29.5	25	2.5

The visual properties of the experimental formulations were evaluated and formulations with appropriate appearance, uniform texture and suitable consistency were selected for other tests. In accelerated stability tests, samples of the selected formulations were submitted to temperature cycles for 28 days (4 °C-40 °C cycles, altering each two weeks). The samples were periodically observed for physical changes such as phase separation, development of objectionable color, odor, and consistency. Centrifugation at 3750 rpm for 15 min was used to measure the accelerated deterioration of the selected formulations. The pH values of the selected formulations, diluted 1:10 in distilled water, were measured at room temperature using a pH meter (CH-8603 model, Mettler-Toledo AG, Switzerland). The most stable formulation with appropriate pH value, which was resistant towards physical changes and centrifugation, was selected as the optimal base for preparation of polyherbal product. All measurements were made in triplicate.

*Preparation of polyherbal ointment*

Based on ITM manuscripts, *M. sylvestris *and *S. nigrum* leaves and *R. damascena* oily extract were used in the proportion of 1:1:4 in burn prescriptions. In the present investigation, we applied the mentioned ratio of the plant materials for preparation of the burn ointment. Since the aqueous extracts of *M. sylvestris *and *S. nigrum* were used in the formulation, the ratio of the plant extracts was modified in the final product. Polyherbal ointment (PHO) was prepared by using 4.85% of each aqueous extracts of *M. sylvestris *and *S. nigrum* and 33% of* R. damascena* oily extract in the best oleaginous base selected from the experimental formulations. 

Briefly, the oily phase consisted of *R. damascena* oily extract, white petrolatum, eucerin and white beeswax was heated in a beaker to about 70 °C using a water bath. After melting of all ingredients, the aqueous extracts of *M. sylvestris *and *S. nigrum*, heated to the same temperature as the oleaginous components, were added to the oily phase and mixed with a stirrer (RZR-2020 model, Heidolph, Germany) at 500 rpm. The mixture was slowly cooled and stirred for 30 min until congealed. BHT and methyl and propyl parabens were used in PHO by adding to the oily phase. 

After checking the visual properties of PHO, physical evaluations were performed through accelerated stability tests, centrifugation and pH assessment. Finally, PHO was submitted to viscosity measurement, microbiological tests and fingerprinting procedure. 

*Viscosity measurement and evaluation of rheological properties of PHO*

The viscosity of PHO was measured at room temperature in triplicate, using a cone/plate Brookfield DV-Ш Ultra Programable Rheometer (Brookfield, USA). 0.5 g of the sample was used in each test. Different shear rates and shear stresses were applied on the sample, and the resulting rheogram was constructed to determine the rheological behavior and viscosity of PHO.

*Microbiological tests*

Microbial limit tests including total viable count (TVC) and tests for specified bacteria (*Staphylococcus aureus* and *Pseudomonas aeroginosa*) were conducted on PHO according to WHO guideline (24). 

*HPTLC fingerprinting of polyherbal ointment*

In order to extract PHO components for high performance thin-layer chromatography (HPTLC), 5 g of PHO was extracted with 2.5 mL of methanol followed by 10 mL water. Aqueous extracts of *M. sylvestris* and *S. nigrum* and methanol fraction of *Rosa damascena *oily extract were used as standard materials. Thin layer chromatography was performed by spotting methanol and aqueous fractions of polyherbal ointment and standards on pre-coated silica gel plate using Camag Linomat 5 automatic sample spotter and 100 µL Hamilton syringe. The samples, in the form of band of length 10 mm, were spotted 10 mm from the bottom using nitrogen aspirator. The development was carried out using mobile phase systems І (toluene: ethyl acetate: acetic acid 60:40:1) and ІІ (ethyl acetate: formic acid: acetic acid: water 100:11:11:10), respectively. At the first, plates were developed with solvent system І to the distance of 185 mm in order to separate less polar constituents. Then they were dried and developed with solvent system ІІ to the distance of 90 mm to detect higher polar substances. Pre-saturation of the chromatographic chamber was performed for both systems for 30 min. The plates were sprayed with NP/PEG reagent (1% methanolic diphenylboryloxyethylamine/5% ethanolic polyethylene glycol 4000). Densitometric scanning was performed on Camag TLC scanner III with Wincats 1.3.0 software (Camag, Switzerland) at 366 nm in the fluorescence mode with slit dimension of 6.00 × 0.20 mm, micro.

## Results and Discussion

*Formulation of a topical preparation for burn wound healing*

The aims of local burn wound management are to reduce pain, prevent pathogens invasion, confirm the integrity of damaged tissue and promote rapid healing with minimal scarring (25, 26). There are three stages for wound healing: inflammation, proliferation and remodeling of the extra cellular matrix. The proliferative phase is defined by angiogenesis, collagen deposition, epithelialization and wound contraction (27). Topically administered drugs are effective in faster wound contraction due to the larger availability at the wound site. The medicaments are dispersed in the base, and later they get divided after the drug penetration into the living cells of skin (28). The importance of moisture for re-epithelialization and angiogenesis is well recognized (25, 29). Recent literatures have been claimed that good hydration is the single most important external factor responsible for optimal wound healing (30). Ointments are typically more occlusive and lubricating than other preparations. Due to the impermeability and soothing properties of the ointments, these dosage forms can be successfully used as suitable bases for topical agents on both partial and full-thickness wounds (31). Oleaginous bases have protecting effect against the escape of moisture and are effective as occlusive dressings. In addition to their emollient effect, these bases can remain on the skin for long periods without drying out (23). Moreover, oleaginous bases may encourage absorption of the medicaments through the skin by improving hydration (32).

In this investigation, a polyherbal ointment was formulated for burn wound healing based on ITM prescriptions. The results of analysis of the plants and their extracts, used in the formulation, have been shown in Table 2. According to the results, the petals of R. *damascena* contained more phenolic compounds and tannins compared to the leaves of *M. sylvestris* and *S. nigrum* while, the aqueous extract of *S. nigrum* appeared to possess the most content of polyphenols and tannins among the examined plant extracts. In order to achieve the best oleaginous base for the product, eight formulations with various amounts of white petrolatum, eucerin and white beeswax were prepared based on construction of a ternary phase diagram. Table 1 shows the composition of base ingredients in the prepared experimental formulations. The attempts were made to investigate the effects of changing the proportion of base ingredients on physical properties of the formulations. The results showed that increasing the proportion of white beeswax, enhanced the stiffness of the formulation which led to a solid texture base (Formulation F_1_). On the other hand, the raised eucerin content (Formulations F_6_, F_7_ and F_8_) or decreased amount of white beeswax resulted in loose consistency of the prepared formulations (Formulation F_5_). Moreover, the glossy appearance of the formulations was increased in accordance with the enhancement of white petrolatum proportion (Formulations F_2_, F_3_, F_4_ and F_5_). By evaluating the visual properties, formulations F_2_, F_3_ and F_4_ presented appropriate consistency and uniformity with suitable glossy appearance. Moreover, these formulations showed acceptable pH values (6.35 ± 0.13, 6.22 ± 0.07 and 6.27 ± 0.11 for F_2_-F_4_, respectively). Due to the optimum visual properties and pH values, formulations F_2_-F_4_ were submitted to accelerated stability tests and centrifugation. Finally, formulation F_3_ exhibited the most stability towards physical changes, so it was selected as the most suitable base formulation for preparation of the polyherbal product. 

**Table 2:** Analysis of *Malva sylvestris*, *Solanum nigrum*, *Rosa damascena* and their extracts

**Plant materials**	**Total ash %**	**Acid insoluble ash%**	**Loss on drying%**	**Swelling index **	**Total polyphenols** *		**Tannins** *	
					**plant**	**Extract**	**Plant**	**Extract**
***M.sylvestris***	15.7±0.1	0.3±0.0	4.9±0.5	11.8±0.3	7.1±0.9	9.1±0.0	1.9±0.1	1.1±0.3
***S.nigrum***	17.6±0.1	2.5±0.2	-	-	9.4±1.2	10.9±0.7	3.7±0.2	1.3±0.1
***R.damascena***	4.5±0.1	-	10.8±1.3	-	34.5±1.2	0.1±0.0	20.4±2.1	0.006±0.002

* *Data expressed as milligram of pyrogallol equivalents per gram plant powder or per milliliter extract.*

Polyherbal ointment (PHO) composed of 4.85% of each aqueous extracts of *M. sylvestris *and *S. nigrum*, *R. damascena* oily extract (33%), white petrolatum (28%), white beeswax (4%), eucerin (25%), BHT (0.04%) and methyl and propyl parabens (0.2% and 0.06%, respectively). The product was glossy brownish green with rose odor. In addition to its appropriate consistency, uniformity and pH value (5.63 ± 0.05), PHO was spread easily on the skin. No signs of phase separation and physical changes were observed in PHO during physical stability tests and centrifugation, thus the formulation was satisfactory with respect to its physical parameters. The results of microbiological tests were consistent with WHO guideline (24). 

*Determining the rheological behavior of PHO*

Nowadays, rheological behaviors including viscous, elastic and plastic properties and combinations of these, viscoelasticity, are considered among the most important and substantial characteristics for semisolids and topically used vehicles (33, 34). Rheology measurements provide a simple and effective means to compare the structural properties of semisolid vehicles (34) and lead to obtain an acceptable formulation with desirable viscosity, stability and durability on the skin surface (35).

**Figure 1:**
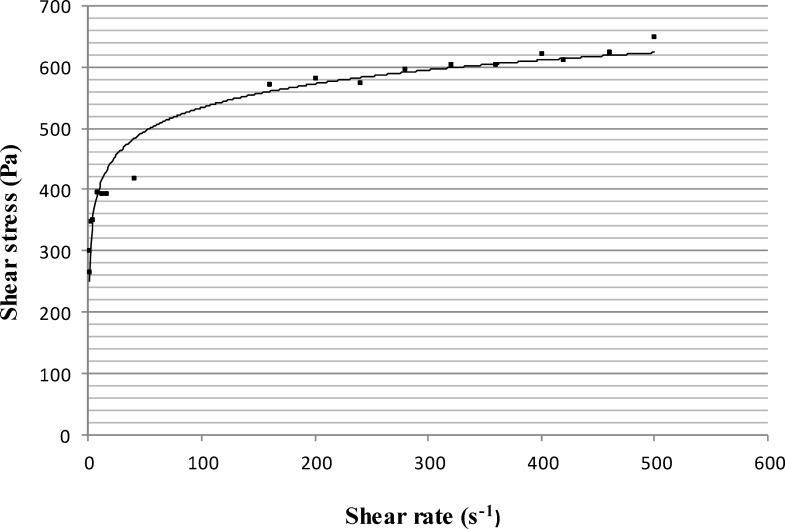
Rheogram of the polyherbal ointment (PHO), showing the presence of a plastic behavior (n = 3, data points are presented as mean ± SD

**Figure 2:**
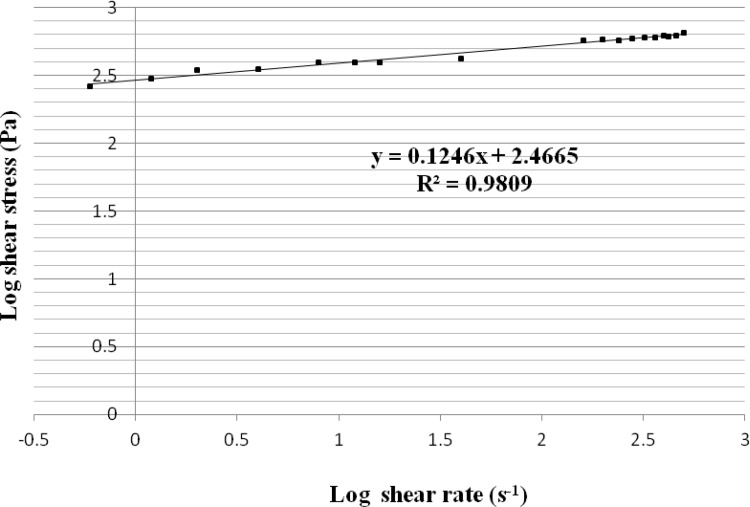
The linear plot of log shear stress-log shear rate for the polyherbal ointment (PHO) (n = 3, data points are presented as mean ± SD

The rheogram of PHO was obtained by using Brookfield stainless steel cone/plate viscometer (Figure 1). According to the figure, the rheogram of PHO was non-linear indicating non-Newtonian behavior. Since the curve did not start from the origin, it can be concluded that PHO showed the plastic (Bingham) rheological behavior. As it turned out, with a more accurate view of the end points, the curve is converted to a line that shows a typical characteristic of plastic behavior which is expected for semisolid products. Regarding to the features of Bingham bodies, it could be expected that the ointment exhibited a yield value. In order to measure the yield value of PHO, the log values of shear stress were plotted against the log values of shear rate (Figure 2). 

By calculating the antilog of y-intercept of the equation (y = 0.1246x + 2.4665) corresponding to the linear plot, the yield value was determined (292.75 ± 15.28 Pa).

**Figure 3:**
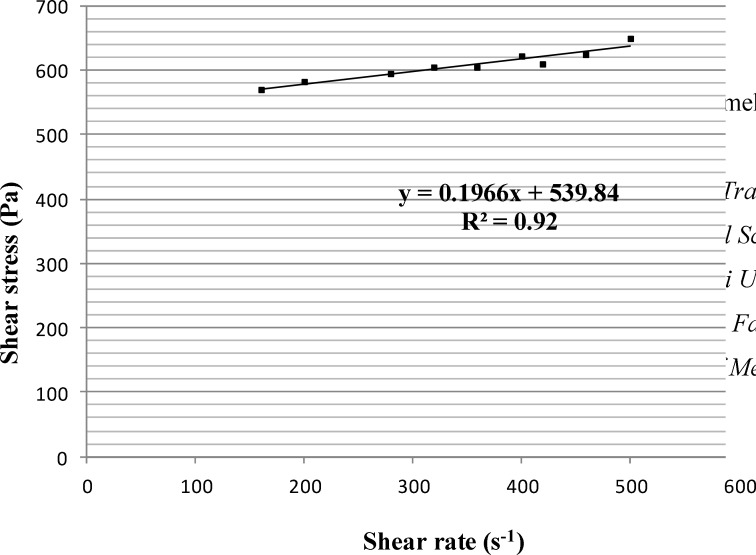
The linear part of the polyherbal ointment (PHO) rheogram (n = 3, data points are presented as mean ± SD

The calculated viscosity and Bingham yield stress of PHO were 0.197 ± 0.086 Pa.s and 539.84 ± 5.47 Pa, respectively, obtained from the slope and y-intercept of the equation (y = 0.1966x + 539.84) corresponding to the linear part of PHO rheogram (Figure 3).

Regarding to the linear part of the rheogram, it was predictable that the amount of Bingham yield stress was greater than yield value indicating the stiffness of the ointment. As PHO was formulated for burn healing, this stiffness brings us to our goal resulting in more durability on the burned tissue.

*HPTLC fingerprinting of polyherbal ointment*

Regarding to the diversity of herbal materials, it is difficult to entirely characterize all these ingredients in herbal products and because of their synergistic effects, identification of the role of each component in therapeutic properties is nearly impossible. Therefore, preserving the quality of herbal products has become an important issue in recent years (19, 20). Qualitative ﬁngerprinting technologies have been used for the quality control of herbal materials as well as herbal preparations lately (19). High performance thin-layer chromatography (HPTLC) is one of the rapid and accurate chromatographic techniques for many phytoconstituents assay and quality control of herbal products. The use of various solvent systems and reagents as well as several development and detection modes, enables HPTLC for parallel and direct comparison of standards with sample components (36). 

**Figure 4:**
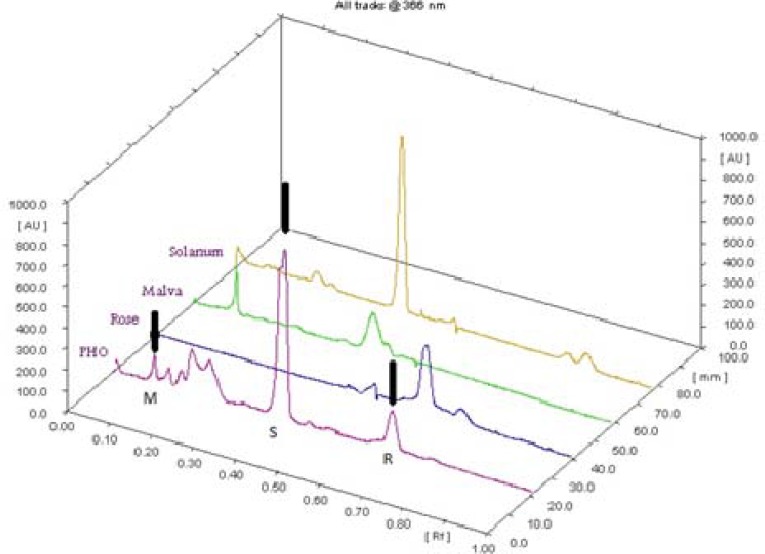
HPTLC fingerprint of the polyherbal ointment (PHO) and the plant extracts. Arrows show the characteristic peaks of the plant extracts in PHO chromatogram (M: Malva sylvestris; S: Solanum. nigrum; R: Rosa damascena

The detection of PHO constituents with different polarities was performed by HPTLC method utilizing methanol and aqueous fractions of the ointment. For spotting the PHO fractions and standard materials, different volumes (10-100 µL) were tested. Finally, volume of 10 µL was chosen for *M. sylvestris* and *S. nigrum* aqueous extracts, while 35 µL was the best volume for PHO fractions (15 µL for methanol and 20 µL for aqueous fractions by loading onto each other). Volume of 70 µL was used for spotting methanol fraction of* Rosa damascena *oily extract. Among several investigated solvent systems, system І (toluene: ethyl acetate: acetic acid 60:40: 1) and ІІ (ethyl acetate: formic acid: acetic acid: water 100:11:11:10) were found to be the most selective and repeatable systems for detecting low and high polar substances, respectively. Development of the plate was performed with systems І and ІІ in two stages which led to detection of less polar compounds in the upper half of the plate and higher polar substances in the lower half. NP/PEG reagent was the reagent of choice by which phenolic compounds and flavonoids appeared in different colored spots (from violet to yellowish-orange under UV light at 366 nm). The HPTLC of the PHO demonstrated the presence of several phenolic and flavonoid compounds which many of them presented similar peaks in the plant extracts profiles. The HPTLC chromatograms of PHO and standards have been shown in Figure 4. The peaks that existed both in the standards and PHO with reasonable heights and good resolution were assigned as "characteristic peaks" for identification of each plant extract in PHO. The chromatograms showed the characteristic peaks of the plant extracts with max R_f_ values of 0.1, 0.41 and 0.66 related to *M. sylvestris*, *S. nigrum* and *R. damascena* extracts, respectively. The chromatogram profiles also demonstrated the specificity of the characteristic peaks of each plant extract in PHO indicating the presence of no detectable peaks in the range of characteristic R_f_ values in other plant extracts.

Plants have extensive potential for the management and treatment of burn wounds with their antioxidant, anti-inflammatory and antimicrobial activities (28). Qualitative and quantitative assay in our study revealed the presence of phenolic compounds and tannins in the extracts used in the polyherbal ointment. Phenolic compounds are thought to be natural sources of antioxidants (37). Flavonoids are considered as powerful free radical scavengers as well (38). It has been claimed that flavonoids influence anti-inflammatory processes by affecting the involved enzymes and pathways (39). Tannins are known as astringent agents. Several studies have demonstrated the antibacterial effect of tannins which could prevent the possible infection during wound healing process (1). Moreover, tannins can precipitate proteins in damaged tissues, resulting in rapid scab formation. This property enables them to decrease tissue edema and exudation along with reducing the permeability of capillaries in the wound (26, 40). It has been claimed that flavonoids and tannins usually influence one or more phases of the healing process through involving in disinfection and debridement and provide a moist and suitable environment for the natural healing process (1). Due to the antioxidant, anti-inflammatory and antimicrobial activities of phytochemical constituents of* Malva sylvestris*,* Solanum nigrum *and *Rosa damascena, *it could be expected that PHO exhibits healing effect on burn wounds in the support of its traditional use in ITM. 

## Conclusion

Based on the Iranian Traditional Medicine (ITM), we prepared a polyherbal topical formulation for burn wounds using leaves aqueous extracts of *Malva sylvestris* and *Solanum nigrum* and *Rosa damascena* petals oily extract which were rich in phenolic compounds. Physical stability and rheological behavior evaluations as well as microbiological tests showed that the prepared formulation was stable with no growth of pathogenic microorganisms and suitable for topical application in burn wounds. HPTLC fingerprinting of PHO demonstrated the presence of several phenolic compounds corresponding to the plant extracts. Moreover, characteristic peaks were observed in PHO profile, so HPTLC fingerprint could be used as an applicable method for quality control of the prepared formulation. Regarding to the role of phenolic compounds in wound healing process, PHO could be an appropriate candidate for burn healing with respect to its traditional use in ITM.

## References

[1] Chaudhari M, Mengi S (2006). Evaluation of phytoconstituents of Terminalia arjuna for wound healing activity in rats. Phytother Res.

[2] Ezzati A, Bayat M, Taheri S, Mohsenifar Z (2009). Low-level lasar therapy with pulsed infrared lasar accelerates third-degree burn healing process in rats. J Reh Res Dev.

[3] Avicenna (2005). al-Qanun fi al -Tibb (The Canon of Medicine). Dar Ehia Al-Tourath Al-Arabi, Beirut.

[4] Jorjani SE (1976). Zakhirah-i Khvarazm'Shahi.

[5] Aghili Shirazi MH (2008). Moalejat-e-Aghili.

[6] Afsharypuor S, Shams Ardekani M, Mosaddegh M, Ghannadi A, Mohagheghzadeh A, Badr P (2013). Introduction to Iranian Traditional Pharmacy and Pharmaceutical Dosage Forms.

[7] Hamedi A, Zarshenas MM, Sohrabpoour M, Zargaran A (2013). Herbal medicinal oils in traditional Persian medicine. Pharm Biol.

[8] Aghili Shirazi MH (2008). Makhzan-ul-Adviah.

[9] Ibn al-Baitar A (1st ed). al-jāmi li-mufradāt al-adwiyawa al-aghdhiya.

[10] Editorial board (2000). PDR for Herbal Medicines.

[11] Barros L, Carvalho AM, Ferreira IC (2010). Leaves, flowers, immature fruits and leafy flowered stems of Malva sylvestris: a comparative study of the nutraceutical potential and composition. Food Chem Toxicol.

[12] Gasparetto JC, Martins CA, Hayash SS, Otuky MF, Pontarolo R (2012). Ethnobotanical and scientific aspects of Malva sylvestris L.: a millennial herbal medicine. J Pharm Pharmacol.

[13] Rhazes. Al Havi (Liber Continent) (2005). Translated by Afsharypuor S.

[14] Heo KS, Lee SJ, Lim KT (2004). Cytotoxic effect of glycoprotein isolated from Solanum nigrum L. through the inhibition of hydroxyl radical-induced DNA-binding activities of NF-kappa B in HT-29 cells. Environ Toxicol Pharmacol.

[15] Abas F, Lajis NH, Israf DA, Khozirah S, UmiKalsom Y (2006). Antioxidant and nitric oxide inhibition activities of selected Malay traditional vegetables. Food Chem.

[16] Shamspur T, Mohamadi M, Mostafavi A (2012). The effects of onion and salt treatments on essential oil content and composition of Rosa damascena Mill. Ind Crop Prod.

[17] Boskabady MH, Kiani S, Rakhshandah H (2006). Relaxant effects of Rosa damascena on guinea pig tracheal chains and its possible mechanism(s). J Ethnopharmacol.

[18] Aghili Shirazi MH (2011). Qarabadin-e-Kabir.

[19] Deattu N, Suseela L, Narayanan N (2013). Chromatographic analysis of polyherbal extract and formulation by HPTLC and GC-MS methods. J pharm res.

[20] Hajimehdipoor H, Kondori BM, Amin GR, Adib N, Rastegar H, Shekarchi M (2012). Development of a validated HPLC method for the simultaneous determination of flavonoids in Cuscuta chinensis Lam. by ultra-violet detection. DARU.

[21] Editorial board (2011). Britsh Pharmacopoeia.

[22] Editorial board (2002). Iranian Herbal Pharmacopoeia.

[23] Allen LV, Popovich NG, Ansel HC (2011). Ansel,s Pharmaceutical Dosage Forms and Drug Delivery Systems.

[24] World Health Organization (1998). Quality control methods for medicinal plant materials.

[25] Singer AJ, Brebbia J, Soroff HH (2007). Management of local burn wounds in the ED. Am J Emerg Med.

[26] Li K, Diao Y, Zhang H, Wang S, Zhang Z, Yu B, Huang S, Yang H (2011). Tannin extracts from immature fruits of Terminalia chebula Fructus Retz. promote cutaneous wound healing in rats. BMC Complemen Altern Med.

[27] Nayak BS, Pereira LMP (2006). Catharanthus roseus flower extract has wound-healing activity in Sprague Dawley rats. BMC Complemen Altern Med.

[28] Thakur R, Jain N, Pathak R, Sandhu SS (2011). Practices in Wound Healing Studies of Plants. Evid-Based Complemen Altern Med.

[29] Demling RH, DeSanti L (2002). The rate of re-epithelialization across meshed skin grafts is increased with exposure to silver. Burns.

[30] Atiyeh BS, Gunn SW, Hayek SN (2005). State of the Art in Burn Treatment. World J Surg.

[31] Ward RS, Saffle JR (1995). Topical Agents in Burn and Wound Care. Phys Ther.

[32] Collett DM, Aulton ME (1990). Pharmaceutical practice.

[33] Mortazavi SA, Pishrochi S, Jafari azar Z (2013). Formulation and in-vitro evaluation of tretinoin microemulsion as a potential carrier for dermal drug delivery. Iran J Pharm Res.

[34] Korhonen M, Niskanen H, Kiesvaara J, Yliruusi J (2000). Determination of optimal combination of surfactants in creams using rheology measurements. Int J Pharm.

[35] Mortazavi SA, Tabandeh H (2005). The influence of various silicones on the rheological parameters of AZG containing silicone-based gels. Iran J Pharm Res.

[36] Hajimehdipoor H, Shekarchi M, Pirali Hamedani M, Abedi Z, Zahedi H, Shekarchi M, Goharie AR (2011). A Validate HPTLC-Densitometric Method for assay of Aucubin in Vitex agnus-castus L. Iran J Pharm Res.

[37] Baydar NG, Baydar H (2013). Phenolic compounds, antiradical activity and antioxidant capacity of oil-bearing rose (Rosa damascena Mill.) extracts. Ind Crop Prod.

[38] Thang PT, Patrick S, Teik LS, Yung CS (2001). Anti-oxidant effects of the extracts from the leaves of Chromolaena odorata on human dermal fibroblasts and epidermal keratinocytes against hydrogen peroxide and hypoxanthine-xanthine oxidase induced damage. Burn.

[39] de Sousa Araْjo TA, Alencar NL, de Amorim ELC, de Albuquerque UP (2008). A new approach to study medicinal plants with tannins and flavonoids contents from the local knowledge. J Ethnopharmacol.

[40] Mendonça FAS, Junior JRP, Esquisatto MAM, Mendonça JS, Franchini CC, dos Santos GMT (2009). Effects of the application of Aloe vera (L.) and microcurrent on the healing of wounds surgically induced in Wistar rats. Acta Cirúrgica Brasileira.

